# Diathesis in Its Relations to Epidemic Diseases

**Published:** 1855-09

**Authors:** J. P. Hall

**Affiliations:** Glasgow, Ky.


### MEDICINE AND SURGERY.

SEPT., 1855. NEW SERIES. VOL. IV.—NO. 3.

### ART. I.—DIATHESIS IN ITS RELATIONS TO EPIDEMIC DISEASES.

BY J. P. HALL, M. D., OF GLASGOW, KY.

WHILE I most earnestly desire to avoid the frivolity of mere medical guess-work, I shall stand unpledged to a strict observance of the rigid but sound and rational Baconian formula as promulgated by Dr. BARTLETT in his philosophy of medical science—"the classification and arrangement of (observed) phenomena and their relationship"—constitutes the fundamental rule in this system, while the Doctor strenuously repudiates every method of scientific inquiry not strictly founded upon observation and experience, or in conformity with the above proposition. Certainly the most if not the only trustworthy line of investigation to be pursued in prosecuting a scientific inquiry is, from "the better to the less known," but unquestionably many of the facts, phenomena and events, constituting materials for the construction of every absolute science, have been wrought from the hidden ore by a less exact process. Hypothesis, speculation, and mere theorizing have, by their direct exercise, resulted in the establishment of no ultimate medical doctrine, yet they serve as valuable suggestive aids in conducting the inquirer onward. And while a large proportion of the laborers in this field are loudly proclaiming the absurdity of medical progress independent of actual observation, they still persist in conjecturing and speculating, and often, as I verily believe, to the advancement of medical truth.

When from an ascertained fact we make a deduction, we have not a second fact till the correctness of our deduction has been verified by experience—we then have a second datum as sound as the original. Suppose we have not a first fact, may we not substitute hypothesis as a basis of deduction, and in like manner subject this deduction to the test of experience? The discovery of this fact is certainly not due to observation alone—this has only confirmed the correctness of that which was brought to view by another process.

Although this article may savor somewhat of speculation, I trust I shall be able to indicate, if not substantiate, some facts which may prove of future value to medical art and science.

As the terms 'epidemic' and 'diathesis' have not uniformly been employed without some degree of vagueness, I may as well, in the first place, designate the conditions which are to be implied by their applications as related to the subject of this paper. In its largest signification I would characterize an "epidemic as such a disease as prevails at certain periods in a marked degree, and is at other periods as markedly absent." But for my present purpose this needs some qualifying explanation. There is a class of zymotics for the production of which there must be a specific internal condition, as well as a specific external condition, and the internal condition exists independently of exterior abnormal circumstances; the action of an external material upon the internal element developes a given process which rids the subject of his former susceptibility, or confers permanent immunity from the self-same disease. This is a class of epidemics to which the views advocated in this paper have no reference. It treats of epidemics that acquire their non-specific internal condition in consequence of the operation of exterior agencies, and which, after the explosion that ensues upon the application of a specific external material, confer but an indefinite immunity upon their subjects.

By way of conveying as clearly and as comprehensively as may be the idea which I shall attach to 'diathesis,' and the relations it has to other actions and conditions, I will attempt to define it by constructing a sort of diagram or formula of propositions: A represents the primary and general vitiating agents; B the healthy human organism; C the primary abnormal state; D the special or specific material; E the individualized epidemic specimen. A, by its action upon B, converts the true norma of B into a state of abnormity, C; while D, by affecting certain relations with C, gives rise to the existence of E.

Now I propose to discuss what I have chosen to denominate the epidemic diathesis, in its relations to the etiology and pathology of epidemic cholera, with such observations relative to dysentery and its congener, typhoid fever, as may serve the purposes of illustration.

It is most obvious if an inquiry of this character is to be prosecuted with any degree of probability that useful results will be attained, that we must endeavor to comprehend, first, the nature of those primary instrumentalities from which given sequences flow; the conditions, special and general, under which such instrumentalities develope such given consequences, both as to the object in operation and the subject operated upon, and also the exact results to which these movements have given rise collectively.

In every instance where the system is brought under the dominion of a morbid diathesis, there is a juncture of time which precisely corresponds to the earliest abnormal impulse excited in the human organism, there is a period which accurately dates the first interference that is had with the true norma of the constitution, and there are special and general agencies which operate, in individual instances, under given conditions to produce these primary impressions. And unless we can gain some real knowledge of the true nature and producing causes of the diathesis, under which epidemic maladies are engendered, we can only learn that which accidental and empirical observation discloses.

In a recent number of the New York Journal of Medicine Prof. Knapp, of Covington, Ky., has published some very interesting observations upon the cause, nature, cure, and prevention of epidemic cholera, in which, he undertakes to establish, that this disease is a correlative of scorbutus. The grounds upon which he bases his arguments in support of his peculiar views, will constitute one branch of the subject of this essay. In this exposition of Dr. KNAPP's views, in reference to a subject of such incomparable moment, there is matter, as I conceive, worthy of most serious and deliberate consideration.

The subject of epidemic etiology has, through all time, been involved in most profound mystery. We lived through a past season without experiencing even an epidemic panic—the following season brings with it no appreciable meteorological dissimilarity, the circumstances in which we exist are in no notable degree altered—yet, epidemic cholera, dysentery, or its legitimate ally, typhoid fever, decimates, aye, dimidiates a community in some instances. These events are the results of laws, and it may be that those laws are of uniform operation, yet so multiform, diverse, and hidden are the elements which enter into their constitution, and under so many apparently conflicting conditions do these elements conspire to produce ultimate results, that the most enlightened research has hitherto failed to remove the veil of obscurity which surrounds the origin of epidemic and endemic diseases. Nevertheless, I venture to assume two propositions—first, that the existence of an epidemic diathesis is an invariable and essential condition to the final developmental process; and secondly, that this is a generic condition. In the next place we inquire as to the causes from which this condition may arise. Telluric and meteorological agencies on the one hand, and dietary on the other, by their conjoint and under their variously-modified aspersions are, as I conceive, fully adequate to the entire process of developing it—more than adequate but for the corrective influence being constantly displayed by a modified form of electricity. M. SCHOENBEIN, whose valuable labors have resulted in the discovery of an agent which he denominates *ozone* has, if his conclusions be correct, accomplished an important step towards a scientific elucidation of this subject.

According to Mr. FARADAY, this extraordinary element which he regards as an allotropic condition of oxygen, but which its discoverer regards as an independent body or condition of matter, forms by weight one-fifth of the atmosphere, and is probably the most powerful oxydizing agent in nature. Cosmic changes, in constant process around us, during the hot vegetating season, would speedily deteriorate the atmosphere to a degree incompatible with health, if not life, but for this depurating agent. I will give an abstract of SCHOENBEIN's conclusions upon one or two points: "The abundant source of gaseous matters is that which the decomposition of vegetable and animal substances affords. Some of these are well known, such as carbonic acid and ammonia; others are of unknown chemical nature, and though the absolute quantity of such deleterious matters may be small in comparison with the immense volume of the atmosphere, their accumulation would render the air unfit for the support of animal life, unless some agent were at work to neutralize or decompose them. For this task ozone is peculiarly fitted by its high oxydizing powers. The purifying influence of thunder-storms upon the atmosphere, is due to the generation of this agent." Thus, while there is an essential necessity connected with the revolutions of generation, growth, and decay, constantly going forward in nature's laboratory, for the evolution of elements most detrimental to our well-being, the great Author has not failed to provide an antidote, with powers commensurate with the magnitude of the evils, which it is its office to counteract. Palludal emanations and effluvia arising from the decomposition of animal substances doubtless constitute by far the most important sources of atmospherical pollution, but it is equally certain that meteorological changes contribute materially to affect its healthful or noxious conditions.

It needs no arguments to establish that these matters exert a most powerful vitiating influence upon the human constitution, I will therefore pass on to a consideration of diet in its causative relations to the epidemic diathesis. If, in pursuing the topics involved in this branch of the inquiry, I shall seem to encroach upon the peculiar domain of Prof. KNAPP, such concurrence of opinion, upon my part, will be received as only corroborative of views already promulgated by him.

So far as concerns the induction of this diathesis, I am not indoctrinated into the Doctor's faith, but in respect to its identity with or very near kindredship to the scorbutic disposition, I entertain but little doubt. I hold that diet only exercises an instrumentality in common with such other agencies as I have and will notice. I am disinclined to believe that any practitioner who has exercised that degree of clinical scrutiny which it behooves every judicious observer to practice, could have failed to detect, in very many instances of cholera, dysentery, and kindred epidemics, in their earlier stages, unmistakable evidences of the scorbutic taint. Although it is but recently that my attention has specially rested upon this feature of these cases, yet, by recurring to the history of many cases, belonging to the family of epidemics alluded to, which have passed under my observation during a period of ten years, I am wholly satisfied that the circumstance is not new, and I am equally satisfied that each consecutive year, during that interval, has stamped the impression more deeply.

The interior of the mouth is the chief seat of these indicative appearances; swollen, spongy, and livid gums, fetid breath, etc., are the indications present as the diathesis becomes confirmed, and during the early stage of the case, purpureous spots or eczemas, and depraved ulcerations as the cases become more advanced.

As to the relations which may subsist between these phenomena and scurvy, and as to the influence which diet may exert in their production, are inquiries of prime interest just now, and such, too, as in reference to which pathologists are likely to hold widely variant opinions.

By a sort of general consent the medical mind of this country seems to have become possessed of an opinion that the scorbutic reign is completely passed, and this is doubtless very true as respects marked cases of scurvy, but as a complication or underlayer of our more malignant epidemics, although a foreigner it still reigns even in this country as well as in its original native land.

Notwithstanding the profession seems thus, in a great degree, to have lost sight of the disease as a subject of separate investigation, yet from European and American medical periodical literature, extending over a period of five years, I could glean quite a mass of observation having an important incidental bearing in confirmation of the doctrines I hold upon this subject. But as I am not disposed to occupy space with extracts, I will merely condense some of the leading views entertained by Dr. BARNES, of the London Hospital, concerning the relations of the condition in question to other morbid conditions. The Doctor deposes very strongly.

He concludes that marked cases of scurvy are not, perhaps, numerous in London, but minor degrees of the scorbutic disposition may be detected on careful inquiry and observation. Patients so affected present themselves at the hospitals complaining of various ailments, etc.—the scorbutic taint being masked by the more prominent disease. Dr. Barnes regards it as certain that if these more prominent diseases have not in all cases arisen as secondary affections upon the scorbutic degredation of the blood, yet that their nature and cause are so modified by this complication that it is necessary to take the scorbutic taint into consideration in prescribing the treatment.

He further states, and still more in point: It is well known that the deaths of thousands of soldiers, registered as owing to variously-named diseases, are in reality to be ascribed, if we ascend to the primary pathological conditions, rather to scurvy—a condition upon which the final and immediately fatal diseases are but epiphenomena. It is obvious enough that Dr. Barnes is smartly imbued with the scorbutic doctrine, and I could introduce numerous other equally respectable witnesses to the same end.

Our advances toward a trustworthy pathological diagnosis, are often materially promoted by attending to the peculiar action of remedial substances, and in reference to no morbid condition, is this, perhaps, more strikingly exemplified than is the case in respect to scorbutus. If we admit evidence upon the principle that under like conditions similar agencies develope identical results, I shall address myself to the task of establishing the proposition not without some encouragement. That the blood has become altered in respect to the normal relations of its primary elements to constitute the scorbutic diathesis, is a proposition too well established to admit of controversy, and that certain alimentary compounds and chemical principles have an intimate relation to this fluid in its development, is also a well fortified proposition. The most potent of these substances, both as to prophylactic and remedial efficacy, are such as contain a large equivalent of acid—esculent vegetables and succulent fruits, contributing both in consequence of their acid constituents and nutritive principles to preserve the blood from scorbutic degradation.

Men of professional sagacity are beginning everywhere to discover the former error of prohibition in the use of fruits and vegetables, as dietetics in epidemic seasons, not only allowing such articles liberally but insisting upon the dangers of abstinence.

Dr. BARNES again says: "That the children at the well-known school at Tooling were mostly disposed to scurvy from bad diet before the cholera broke out among them."

Dr. BUDD, whose judgments are ever deliberate and sound, in concluding some clinical remarks in relation to a case of scurvy, thus indicates his opinions upon this topic: "This case shows very forcibly that total abstinence from vegetable food is very likely to give rise to scurvy, and may serve as a warning, should cholera unfortunately visit us again, to those who, from fear of the epidemic, carefully avoid fruits and vegetables. Exclusion is not the proper system to follow; moderate use of animal and vegetable food, open air, healthful labor and exercise, and the avoidance of any kind of excess, will be trustworthy prophylactics of both complaints."

Our best collective data are made up from individual experience, and we have the concurrent testimony of a sufficient number of individual members of the profession to the prophylactic and therapeutic value, under these tendencies of the system, of various vegetable and mineral acids to establish their claims upon a high basis. We have abundant medical record to the effect that the inhabitants of cider districts in various portions of Europe have enjoyed an almost entire immunity from cholera, otherwise no more blessed in point of hygienic protection than immediately neighboring communities, who have suffered most direful visitations. I presume the experience of almost every enterprising practitioner has tested the validity of the claims of the acid treatment in a certain class of epidemic cases. Citric acid, which, since the earliest records of medical experience, has enjoyed favor superior to almost any other substance in correcting scorbutic cachexy, I have used in the special class of epidemics under consideration with the most decided good results. Sulphuric and nitric acids—the latter being the great Austrian remedy in cholera—have become leading and standard remedies everywhere in treating such varieties of affection as are characterized by a bowel profluvium.

There is at this time prevailing in this portion of the country a very malignant type of dysentery characterized by adynamic fever, extreme impoverishment and depravity of circulation, etc., which I have, more recently, treated by literally 'acidulating' my patients and with evidences more conclusive of benefit than have resulted from any plan I have been able to devise.

My friend Dr. Rogers, of this place, a man of mature experience, related to me, a few days since, a circumstance of singular significance, connected with an extreme case of typhoid fever which became the subject of his clinical care. The subject was a middle-aged, laborious agriculturist, and the disease had progressed to an extremity so critical that the Doctor despaired entirely of his patient's recovery. For several days his patient had most importunately teased him for permission to drink cider. This permission was persistently withheld till the case grew so hopeless that it was thought matters could be made no worse, and the cider was allowed to complete satiety. The sequel is that the sick man at once convalesced, and without any more physical favors from the Doctor, he speedily recovered his health entirely.

My particular friend and neighbor, Dr. Trabue, informs me that during the prevalence of the present epidemic of dysentery, he has allowed such patients slightly acid cider *ad libitum*, with results so beneficial as to inspire him with great confidence in its value as a dysenteric remedy.

I could adduce facts of this character to any extent that the positions I have undertaken to vindicate might seem to need such corroboration, but I can feel no hesitancy in coming to the belief that no man of medical experience, who will subject his own observations to a critical analysis, can withhold his convictions aside from any force which may attach to the foregone facts and arguments.

I have thus far attempted to show that the existence of a diathesis is an essential condition to the ultimate disease—developing impulse; that this condition is generic; that vegetable and animal miasma, together with the hygrometic and thermtic state of the atmosphere, on the one hand, and unwholesome or deficient alimentary supplies on the other, are fully adequate to the induction of such a morbid proclivity in the constitution.

I will now offer some suggestions as to the derivation and causative relations of the specific element, in consequence of the action of which, in view of the above permissive state of the constitution, characteristic morbid expressions ensue. Whether the general causes which have been treated of as capable of abnormalizing the system into a diathesis, become, under any circumstances, sufficiently intensified to originate cholera independent of a self-generated medium, is a question which must be determined upon very nice data. From a limited individual experience and a tolerably extensive examination of recorded testimony bearing upon this point, I am decidedly favorable to such an idea, though by no means to the exclusion of the view that a specific product is the immediate agent.

And while I combat the idea that the disease is in strictness contagious, I hold it to be self-propagating and portable, but the portable material not at all times derived from the same source, generated from the same elements or by a like process. I think that we may confidently infer the existence of a specific product as the only *immediate* agent in the production of Asiatic cholera, and in seeking for the sources of this poison, and the agencies which conspire to effect its elaboration, we look to the matters eliminated from the human organism for the first, and solar and atmospherical influences as the second. We have every reason to conclude that a perfect coaptation of every circumstance essential to this result, is of very unfrequent occurrence and short duration, otherwise we should not witness that erratic course which uniformly characterizes the epidemic. The alvine excreta, after having been deposited (in the temples of Cloacina), being subjected to the influences, already alluded to, doubtless constitute a most prolific source of this specific material, although the bodies from which they may have been elimenated were in a normal state or afflicted with unlike maladies. But the prime source of the origin of this virus in its fatal abundance and most deadly concentration, is the chemical changes which occur in the true choleraic eliminations.

Certain conditions of the atmosphere, temperature, moisture, electricity, etc., may be present to day in most effective force for the chemical changes necessary for the evolution of this deadly matter, but to-morrow the ozonometer changes, and pestilence ceases to "walk by night" till the original conditions for evil are restored. The greater the accumulation of such matters in a given area, as for instance populous cities, *cæteris paribus*, the more abundantly must the cholera virus be liberated.

A single case of cholera imported into a large city, the inhabitants of which are, in large proportions, already laboring under a well-established epidemic diathesis, may, in conformity with the above view, originate and propagate the epidemic in its most direful form, and yet display not a property of contagion. But if the inhabitants of such city had escaped the primary abnormalizing influences, and the coaptation of other named circumstances was wanting, the attempt to propagate and introduce cholera by such a means, would amount to sowing seed in dry ground; the disease would not linger one hour beyond the termination of its career in the solitary imported case. We hear much said about 'ochlitic miasm,' a disease—producing element, generated by crowding the sick together: this is doubtless a potent agent in developing cholera, but its origin is animal excretions and exhalations, and the identical conditions, of which I have been speaking, must conspire in its production and dissemination to endow it with *essential* specific properties. It is therefore the self-same agent to which I *invariably* ascribe the *immediate* development of the disease.

The contagion theory, for all that I know to the contrary, affords no satisfactory explanation of the propagation of cholera, while the above view approximates to an explanation of every difficulty that has presented itself to my mind.

The entire problem, in my view, resolves itself into this: Certain substances or particles of matter of animal origin mainly, if not exclusively, subjected to certain chemical laws (atmospherical media), develope given processes upon which is resultant a spespecifically endowed poison.

Now as to the animalcular theory, the astral, and innumerable other fanciful and extrordinary theories, I hold them to be simply preposterous, and would willingly co-operate in repudiating the entire batch.

In a very brief manner I will offer what I conceive to be some rational pathological deductions.

Asiatic cholera is characterized by a group of phenomena which manifest themselves, usually, in very regular order. Passing by the period prior to its open manifestation, we have first flocculent rice water diarrhœa and vomiting, cyanosis and algidity, cramps, asphyxia and coma, suppressed functions, etc. The rationale of these phenomena has been attempted by very elaborate arguments based upon numerous and widely-antagonistic grounds. Some of these theories are most complex, while others are so simple as to "vitiate themselves by offering a too easy explanation of an intricate problem."

The neurotic theory, or the one referring the essential seat of cholera to the solar plexus and its appendages, or the sympathetic system of nerves is, just now, more than any other in vogue. The more important considerations insisted upon by the vindicators of this doctrine are these: "That neither the symptoms nor the postmortem evidences indicate the presence of any diseased organic action proceeding within any separate tissue or organ, and that the whole system becomes affected at about the same period." To my mind this is all susceptible of a more satisfactory solution, without disturbing the repose of the nervous system, for such an explanation as refers difficult problems to this system is, at best, but rendering the mysterious more mystic.

As to the toxical doctrine, which ascribes every pathological manifestation to the diffusion of a subtle poison, I have not, in the least, been impressed with its plausibility. I am a decided advocate for the poison (specific), and equally so for the doctrine which refers the essential seat of the disease to the blood. The blood being already imbued with certain affinities, the introduction of the true specific results in the separation of the aqueous and saline particles from the solid materials of the blood. This constitutes a primary and essential feature of the disease. The next movement is evacuation of these vascular contents by exosmosis into the intestinal tract. Whether the further evacuation of these sanguineous components from the bowel is accomplished or not, is immaterial as to the effects upon the general functions of the economy, the remaining circulatory medium has lost its physiological characteristics and ceases its physiological offices, and from this as a primary condition do all other and secondary conditions arise. The blood thus altered in its hystological relations is not only deficient in capacity for vital stimulation and normal plasticity, but it has become mechanically and vitally disqualified for permeating those structures whose functional integrity and those tissues whose preservation and growth are due to its energizing presence. The vascular current, without the life-giving ministrations of which every organic force must cease, has become an inspissated, grumous, devitalized mass.

Under these circumstances should we be surprised that the functions of assimilation, nutrition, secretion, calorification, innervation, and finally, respiration, and the heart's action, are become suspended? More especially have we little reason to be astounded at the supervention of ganglionic paralysis, even a state of anesthesia, with a motionless mass of carbonized blood to subserve the wants of a constantly changing organism. It is not a subject for wonder either that algid cholera is a fatal disease—it is essentially so, to be otherwise we have the greatest cause for astonishment. Molecular death has already taken place, and but too surely and speedily must somatic death ensue. Every condition upon which the contiunance of vitality depends has become undone.

Although I regard the separation of this train of extreme events as wholly impracticable, yet the thwarting of those agencies which, in the progress of their actions give rise to such events, I regard as clearly within the scope of successful medical interference.

The original diathesis is preventable upon the plain prinicples of medical hygiene, involving a rigid application of such rules as are deducible from the nature of causes and their effects. The special infection or cholera virus is derived from sources and engendered under conditions in reference to which we may attain to such knowledge as will enable us to solve the most important problem in the whole category. When the disease has assumed a manifest semiology, a timely application of our art, under a judicious eclecticism from its healing stores, will but rarely fail in staying progress prior to the attainment of that stage which is without hope.

In view of these facts, if they are accepted as such, it behooves every medical philanthropist to contribute his aid in giving the fullest efficiency to such sanitary efforts as are most likely to dissipate the earlier as well as the more immediate causes of this direful malady, and if such efforts fail in their prophylactic ends, the most prompt, decided, and efficient instrumentalities of arrestment must be employed as the *ultima thule* of our hopes.

